# Activity of the Tobacco Industry in Research and Scientific Literature

**DOI:** 10.1177/1179173X241271566

**Published:** 2024-08-19

**Authors:** Markus Braun, Doris Klingelhöfer, Dörthe Brüggmann, David A. Groneberg

**Affiliations:** Institute of Occupational Medicine, Social Medicine, and Environmental Medicine, 9173 Goethe University Frankfurt, Frankfurt am Main, Germany

**Keywords:** tobacco companies, research output, conflicts of interest, big tobacco, funding, interest-led research support

## Abstract

**Introduction:**

Tobacco companies conduct and fund research. They are not always interested in open-ended research. They promote their interests through public relations campaigns. It’s a proven fact that they influence the scientific community by impairing scientific reputation, especially in the case of health-related research.

**Methods:**

To obtain a comprehensive picture of research and funding activities of the tobacco industry as well as studies about the tobacco industry, respective scientific articles were analyzed in terms of temporal aspects, research areas, networking, and funding sources using established and advanced bibliometric methods.

**Results:**

We found the foci of publications with tobacco industry involvement or funding were mainly in chemistry, toxicology, pharmacology, and agricultural sciences. Health-related scopes occurred much less frequently. In contrast, health and medical sciences were the main focus of publications on the tobacco industry. The Chinese state-owned CNTC was the most research-involved tobacco company and often networked with Chinese academic institutions. Whereas, Western universities, on the other hand, collaborated with tobacco companies to a much lesser extent.

**Conclusion:**

Conflicts of interest of researchers or academic institutions with the tobacco industry occur repeatedly. That is highly problematic and should not be ignored by the scientific community. The science and the public should be skeptical about tobacco industry-supported research.

## Introduction

As early as 1939, two surgeons and medical researchers, Alton Ochsner and Michael DeBakey, published their groundbreaking paper on the association between smoking and carcinoma of the lung,^1^ confirmed in a landmark article in *The Journal of the American Medical Association* (JAMA) in 1950.^2^ In the 1960s, the Framingham Heart Study found cigarette smoking increases the risk of heart disease.^3^ The US Surgeon General’s 1964 report on smoking and health highlighted the health hazards of smoking and was a great media event.^4^ In the early 1950s, the tobacco industry started to undermine the scientific findings and evidence of the health risks of smoking by elaborate PR moves intending to impeach the credibility of science.^5^ The Chief Executive Officers (CEOs) of the biggest US tobacco companies and the head of a powerful PR company, John W. Hill, established 1954 the “Tobacco Industry Research Committee”, later called the “Council for Tobacco Research” (CTR).^5,6^ Hill convinced the CEOs that it would be better to support science than to shun it and do science to sow doubt. Their strategy aimed to amplify the skeptics of the link between smoking and diseases, among others, by funding science, creating controversies in the scientific community.^5^ They wanted to provide evidence to convince smokers that they have nothing to fear and to reassure the public, particularly smokers.^7^ After the dissolution of the two industry-wide research programs, CTR and Center for Indoor Air Research (CIAR), the research grant program “Philip Morris External Research Program” seemed to restore the scientific credibility of the tobacco industry.^8^ As late as 1999, tobacco companies maintained the stance that the health hazards of smoking are not proven, although some of their scientists held contrary views not made public.^9^ In 2020, at the beginning of the COVID-19 pandemic, news items based on preprints reported worldwide that nicotine protects against COVID-19,^10,11^ meanwhile disproved. There were connections between the study group of the underlying publications and the tobacco industry.^12^ The tobacco industry repeatedly seeks to undermine, influence, and misuse science to its advantage at public health expense.

To what extent and in what ways has the tobacco industry influenced the research landscape, and has anything changed over time? We sought to address this question by conducting bibliometric analyses of the published scientific studies where authors were affiliated with the tobacco industry, studies funded by the tobacco industry, and studies about the tobacco industry. The presented findings of this study enable the interpretation of trends, focal points, and linkages in tobacco industry-related research and their significance for future research projects.

## Methods

### Methodological platform and data source

This study uses the methods of the established bibliometric platform *New Quality and Quantity in Science* (NewQIS).^13^ NewQIS provides tools for analyses of publication metadata on scientific topics. For this purpose, an internal project algorithm was created, which collects the metadata and stores it in an MS Access database. The database generated in this way is the basis for all further analyses.^13,14^ The standard data source for scientific literature is Clarivate’s online database *Web of Science* (WoS), one of the largest online scientific literature databases and well established in bibliometric analyses. WoS lists publications from peer-reviewed and quality journals with their annual citation counts from 1900 to the present.

### Search strategy and data processing

Three focal points were formed for the evaluation examining the scientific performance concerning the tobacco industry in more detail: search area A) = S_Affiliations_: publications of tobacco industry affiliated authors (at least one); search area B) = S_Funder_: publications funded (at least partly) by the tobacco industry; search area C) = S_Title_: publications about the tobacco industry.

The following search strategies were used, which combined names of all relevant tobacco companies worldwide and the associated terms that uniquely identify them into a detailed string for each focus.

1. Search area A) = Publications from authors affiliated with the tobacco industry = S_Affiliation_: WoS Field = AFFILIATION or ADDRESS. Search string: “tobacco industr*” OR “tobacco compan*” OR “tobacco manufact*” OR “tobacco corp*” OR “tobacco factory” OR “tobacco factories” OR “cigar* industr*” OR “cigar* compan*” OR “cigar* manufact*” OR “cigar* producer*” OR “ cigar* factory” OR “ cigar* factories” OR “Philip Morris*” OR “British American Tob*” OR “China Tobacco*” OR “Zhengzhou Tobacco Research Institute*” OR “CNTC” OR “Imperial Brand*” OR “Imperial Tobacco*” OR “Altria*” OR “Japan Tobacco*” OR “Sampoerna tbk” OR “pt Sampoerna” OR “hm Sampoerna” OR “Swedish Match*” OR “Reynolds tobacco” OR ″R.J. Reynolds” OR “RJ Reynolds” OR “Souza Cruz SA*”
2. Search area B) = Publications funded by the tobacco industry = S_Funder_: WoS Field = FUNDING AGENCY. Search string: Same as for search area A)
3. Search area C) = Publications about the tobacco industry = S_Title_: WoS Field = TITLE. Search string: “tobacco industr*” OR “tobacco compan*” OR “tobacco manufact*” OR “tobacco corp*” OR “tobacco factory” OR “tobacco factories” OR “cigar* industr*” OR “cigar* compan*” OR “cigar* manufact*” OR “cigar* factory” OR “cigar* factories” OR “Philip Morris” OR “British American Tobacco” OR “Imperial Tobacco” OR “Japan Tobacco” OR “Reynolds tobacco” OR ″R.J. Reynolds” OR “RJ Reynolds” OR “R.J.Reynolds” OR “Altria” NOT “D. Altria”

Each string consists of terms linked with the Boolean operator “OR” using asterisks as placeholders for the sequence of the different characters. After the search input, all entries found were filtered for original articles. No time limit was applied, so all years for each focus were included. However, this resulted in different evaluation periods since the funding agencies were systematically listed in WoS only from August 2008 onwards.

For evaluating the institutions involved, it was necessary to standardize their various designations. The companies Philip Morris International (PMI), Philip Morris Companies Inc, and Altria Group have been combined under the term Philip Morris, based in the USA (Corporate Headquarters) and Switzerland (Operations Center), for reasons of company history. The countries of origin of the founded articles have been updated with a current list of countries and regional territories.

The collected and manually standardized data was stored in an MS Access database and sorted for the three search processes (S_Affiliation_, S_Funder_, S_Title_) according to the parameters to be analyzed.

### Data analysis

The analyses were carried out separately for the retrieved metadata of the publications of the three search strategies (S_Affiliation_, S_Funder_, S_Title_) based on the generated databases.

The data were analyzed according to chronological parameters, including annual publications, citation counts, and citation rates. The citation rates were calculated by the quotient of citations per article. Furthermore, the number of articles was related, also by calculation of the quotient, to the number of Science Citation Index Expanded (SCIE) articles to show the relative development of publication numbers. Also, the most cited articles were identified. Another focus was the analysis of the respective research areas. For this purpose, the WoS research categories were analyzed, whereby the most frequently assigned categories were identified, and their proportional change was determined at 5-year intervals.

The publishing institutions of the three search areas were analyzed, which required the standardization and normalization of all assigned affiliations. The so-identified most-publishing tobacco companies were also analyzed regarding the number of articles, citations, and citation rates. Cooperation networks at the institutional and national levels were analyzed. A cooperation article is defined as having at least two affiliations from different countries (national level) or authors from different institutional affiliations (institutional level). Matrices with values of cooperation were computed and transformed into vectors by a developed software module.^14^

The funding of research was another focus of this study. The funding organizations were identified for all three search areas. For this purpose, the sources of financing had to be standardized, the designation of the funds and the separately listed financing numbers assigned to a source organization or a company.

In addition, the publishing journals were analyzed and compared for the three search areas (S_Affiliation_, S_Funder_, S_Title_).

## Results

**Figure 1.**
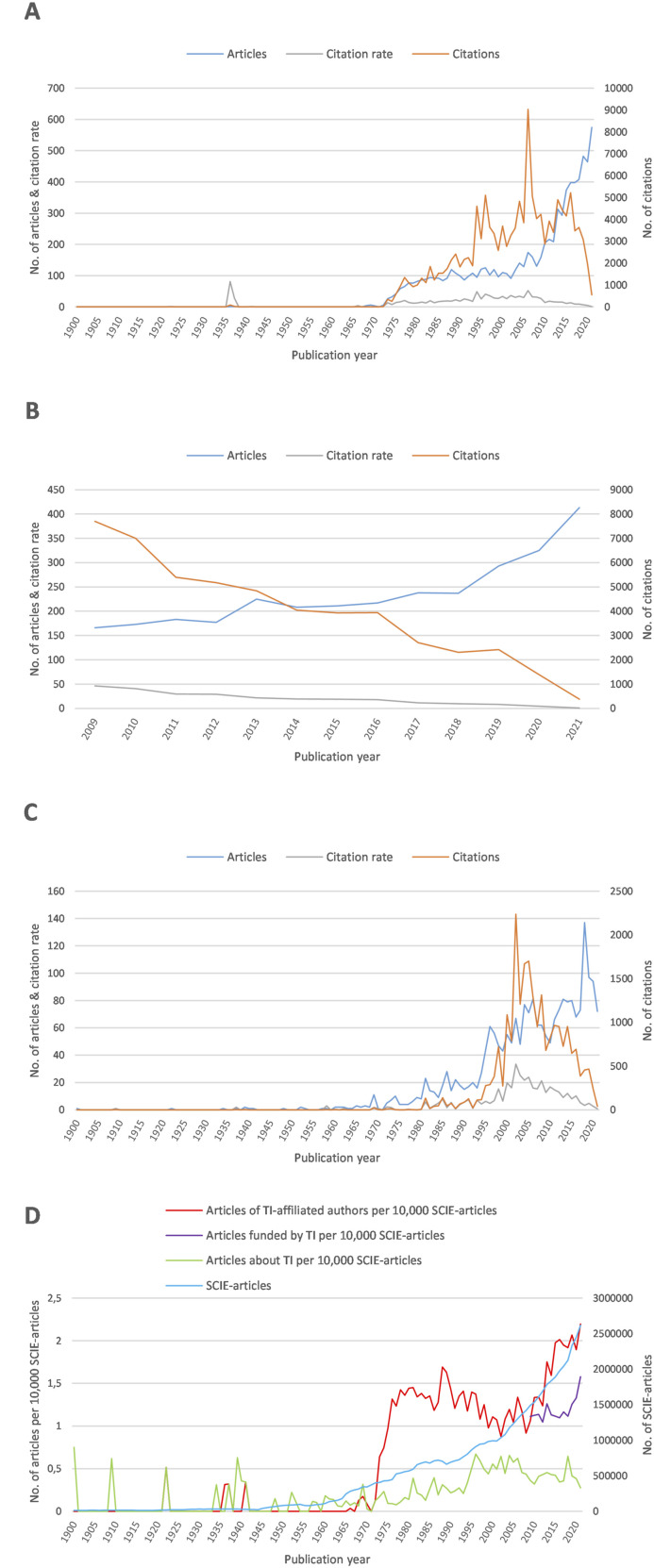
Publication development over time with the number of articles, number of citations, and citation rate. Only completed years are included. A: Articles from authors affiliated with the tobacco industry from 1900 to 2021. B: Articles funded by the tobacco industry from 2009 to 2021. C: Articles about the tobacco industry from 1900 to 2021. D: Relative number of articles (search areas S_Affiliation_, S_Funder_, S_Title_) per 10 000 articles listed in the Science Citation Index Expanded (SCIE) database of Web of Science. TI = Tobacco industry.

In the WoS database, beginning January 1900 until January 2022, n = 8077 articles were found where at least one tobacco industry affiliated author (S_Affiliation_). These articles were cited 138 058 times in this period, meaning a citation rate cr = 17.09. The WoS category “Funding Agency” is available just since August 2008.^15^ From 2008 until January 2022, n = 3186 articles were found funded by the tobacco industry (S_Funder_), which were cited 55 545 times in that period (cr = 17.43). From January 1900 until January 2022, n = 2204 articles about the tobacco industry (S_Title_) were found, cited 23 321 times (cr = 10.58). Figure 1 shows the publication development over time of all three search areas.

### Publication development over time

#### S_Affiliation_

The first article we found by authors affiliated with the tobacco industry, titled ‘Chemistry as an Aid to the Tobacco Industry’, was published in 1922.^16^ The sole author was a chemist of The American Tobacco Company (British American Tobacco, BAT). By 1966, we identified only four more articles published by authors affiliated with the tobacco industry. Between 1968 and 1972, n = 18 articles overall were identified. 1973 was the first year with a two-digit publication number (n = 26). From then on, the number of publications per year increased more or less consistently until 2009 (n = 158 articles). From 2010 to 2021, the slope increased and reached n = 575 in 2021, the peak so far. The annual citations (c) increased abruptly from c = 9 in 1972 to c = 344 in 1973 and continued to rise subsequently to c = 5224 in 2016, with peaks in 1994 (c = 4605), 1996 (c = 5110), 2006 (c = 9031), and decreased afterward to 552 in 2021. The 23 articles published between 1900 and 1972 were cited very sparsely, except for the second and third publication from 1936 and 1937, with c = 81 and c = 28, respectively. The publications from 1966, 1968, and 1972 reached, by contrast, only cr = 4, 0.5, and 1.8, respectively. From 1973 onwards, the citation rate increased from 13.23 to 51.9 in 2006, with peaks in 1977 (cr = 20.71), 1994 (cr = 48.47), and 1996 (cr = 40.88), and decreased then to 1.04 in 2021 (Figure 1(A)).

The annual ratio of articles from authors affiliated with the tobacco industry and all SCIE articles jumped between 1972 and 1976 from far below 1 to 1.32 articles per 10 000 SCIE articles and again in 2013 to 1.75 and further increased to 2.2 in 2021 (Figure 1(D)).

#### S_Funder_

From 2009 to 2021, the number of articles funded by the tobacco industry increased relatively consistently from n = 166 to n = 413 per year, with a slightly steeper rise since 2018. The number of citations decreased steadily from c = 7694 in 2009 to c = 374 in 2021. The citation rate decreased likewise from cr = 46.35 in 2009 to cr = 0.97 in 2021 (Figure 1(B)).

The annual ratio of tobacco industry-funded and listed SCIE articles increased from 1.11 in 2009 to 1.58 in 2021 per 10.000 (Figure 1(D)).

#### S_Title_

The first article identified about the tobacco industry was published in 1900 in JAMA and dealt with tobacco amblyopia - a visual impairment due to tobacco - among tobacco factory employees in Cincinnati, USA.^17^ Through 1968, n = 32 more articles were published, with a maximum of three articles per year (1965 and 1967). 1969 was the first year with two-digit publication number (n = 11), followed by 1974 with n = 10, and 1981 with n = 23 publications per year. From then on, the number of articles per year remained consistent between n = 9 and n = 28 until 1994. From 1995 until 2021, the number increased slowly from n = 44 to n = 72, with little peaks in 2006 (n = 81), 2018 (n = 137), 2019 (n = 97), and 2020 (n = 94). The 33 articles published between 1900 and 1968 were only ten times cited overall. 1969 was the first year with a two-digit number of citations (n = 20), followed by 1972 (n = 11) and 1973 (n = 12). 1981 showed the first peak with n = 133 citations, also 1985 (n = 138) and 1991 (n = 127). From 1993, the number of citations rose from 114 to the highest, 2237 in 2002, followed by four-digit numbers between c = 1208 and c = 1700 from 2003 to 2006 and 2008. Afterward, the number of citations decreased to 42 in 2021. The citation rate followed approximately the run of the citation curve, with the highest peak also in 2002 (cr = 33.39) (Figure 1(C)).

The annual ratio of articles about the tobacco industry per 10.000 SCIE articles fluctuated between 0 and 0.75 (Figure 1(D)).

Table S1 in the supplementary files lists the ten most cited articles in the three analyzed search areas.

### Research areas

**Figure 2.**
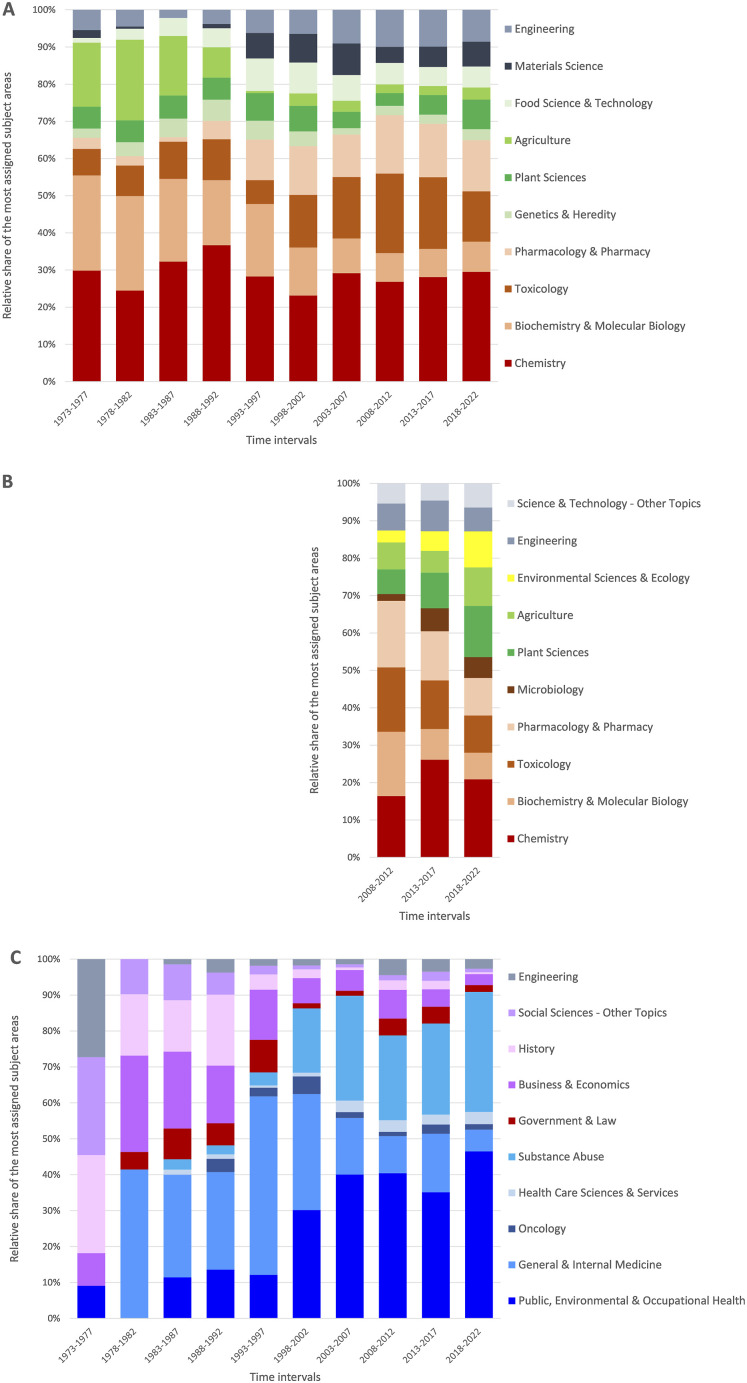
Relative share of the ten most assigned Web of Science (WoS) research areas in 5-year intervals sorted by affinity. A: Articles from authors affiliated with the tobacco industry from 1973 to 2022. B: Articles funded by the tobacco industry from 2008 to 2022. C: Articles about the tobacco industry from 1973 to 2022.

Figure 2 shows the evolution of the ten most assigned WoS research areas in 5-year intervals from 1973 to 2022 (S_Affiliation_ and S_Title_), respectively 2008 to 2022 (S_Funder_). Table 1 lists the 15 most assigned WoS research areas of the three search areas sorted by the number of articles. Additionally, Table S2 in the supplementary files lists all assigned WoS research areas with at least one article sorted by the number of articles.

**Table 1.** Fifteen most assigned Web of Science (WoS) research areas sorted by number of articles of the three search areas (S_Affiliation_, S_Funder_, S_Title_) analyzed with stated percentage of the total number of articles.

WoS Research Area	Articles	Percentage	Citations	Citation Rate
A: S_Affiliation_				
Chemistry	2241	18.7	35258	15.73
Toxicology	1128	9.4	19520	17.30
Biochemistry & Molecular Biology	959	8	22161	23.11
Pharmacology & Pharmacy	872	7.3	12953	14.85
Engineering	599	5	9206	15.37
Plant Sciences	465	3.9	10892	23.42
Food Science & Technology	437	3.7	8616	19.72
Agriculture	432	3.6	5015	11.61
Materials Science	392	3.3	9051	23.09
Genetics & Heredity	251	2.1	4793	19.10
Environmental Sciences & Ecology	241	2	3502	14.53
Biotechnology & Applied Microbiology	235	2	4432	18.86
Physics	198	1.7	5317	26.85
Science & Technology - Other Topics	198	1.7	3937	19.88
Energy & Fuels	160	1.3	5538	34.61
B: S_Funder_				
Chemistry	569	12.4	7001	12.30
Pharmacology & Pharmacy	330	7.2	5776	17.50
Toxicology	319	6.9	5244	16.44
Plant Sciences	274	6	2719	9.92
Biochemistry & Molecular Biology	246	5.3	6759	27.48
Agriculture	204	4.4	1237	6.06
Engineering	187	4.1	2295	12.27
Environmental Sciences & Ecology	172	3.7	2194	12.76
Science & Technology - Other Topics	143	3.1	4080	28.53
Microbiology	131	2.8	1243	9.49
Biotechnology & Applied Microbiology	125	2.7	2407	19.26
Materials Science	122	2.6	2772	22.72
Food Science & Technology	114	2.5	1327	11.64
Neurosciences & Neurology	114	2.5	2727	23.92
Genetics & Heredity	103	2.2	2038	19.79
C: S_Title_				
Public, Environmental & Occupational Health	845	26.7	15079	17.84
Substance Abuse	563	17.8	9904	17.59
General & Internal Medicine	451	14.3	4188	9.29
Business & Economics	185	5.9	1172	6.34
Government & Law	85	2.7	339	3.99
History	75	2.4	51	0.68
Engineering	67	2.1	147	2.19
Health Care Sciences & Services	61	1.9	626	10.26
Social Sciences - Other Topics	53	1.7	293	5.53
Oncology	51	1.6	286	5.61
Respiratory System	44	1.4	73	1.66
Psychiatry	41	1.3	713	17.39
Science & Technology - Other Topics	33	1	156	4.73
Computer Science	32	1	31	0.97
Agriculture	30	0.9	218	7.27

#### S_Affiliation_

The 8077 articles were 11 962 times listed in 120 WoS research areas. Among the ten most assigned research areas (Figure 2(A)), the focus areas were chemical and life sciences and technology and engineering, respectively, taking into account that Chemistry took the highest proportion over the years either more or less consistently. From 1973 onwards, the relative share of the WoS research areas *Biochemistry & Molecular Biology* and *Agriculture* decreased rather distinctly, and that of *Toxicology*, *Pharmacology & Pharmacy*, *Material Science,* and *Engineering* increased more or less. The research areas *Public, Environmental & Occupational Health* (rank 21 of 120) and *Oncology* (rank 25) were the most assigned in the fields of health and medical sciences, with a relative share of barely 1% each (Supplement Table. S2A).

#### S_Funder_

The 3186 articles funded by the tobacco industry were 4605 times listed in 111 WoS research areas. The ranking of the ten most assigned WoS research areas was similar to that of search area SAffiliation. It differed that in place of Genetics & Heredity, Food Science & Technology, and Materials Science, the WoS research areas Microbiology, Environmental Sciences & Ecology, and Science & Technology – Other Topics were among the first ten (Figure 2(B)). Here also Chemistry had the highest share of all listed research areas, and, likewise, research areas from the health or medical sciences played a more of a minor part. Public, Environmental & Occupational Health was the first listed in this area with a share of under 2% (rank 16 of 111) (Supplement Table. S2B).

#### S_Title_

The 2204 articles about the tobacco industry were 3162 times listed in 96 WoS research areas. Among the ten most assigned WoS research areas, compared to the search areas *SAffiliation* and *SFunder*, only *Engineering* was also listed here (Figure 2(C)). Four research areas belonged to health or medical sciences, *Public, Environmental & Occupational Health* (rank 1), *General & Internal Medicine* (rank 3), *Health Care Sciences* (rank 8), and *Oncology* (rank 10) representing 44.5% of all listed articles (Tab. S2C).

### Most publishing tobacco companies

Table 2 shows the most publishing tobacco companies and institutions concerning the three search areas sorted by the number of articles. In the table, the thresholds for institutions were set at 50 articles for search areas S_Affiliation_ and S_Funder_ and at 20 articles for S_Title_. All tobacco companies with at least one article were listed.

**Table 2.** Most publishing tobacco companies and institutions in the three search areas (S_Affiliation_, S_Funder_, S_Title_) measured by the number of articles. CNTC = China National Tobacco Corporation. JTI = Japan Tobacco Inc. PM = Philip Morris. RJR = R. J. Reynolds Tobacco Company. BAT = British American Tobacco.

Tobacco Company / Institution	Country	Articles	Citations	Citation Rate
A: S_Affiliation_: List threshold at least 50 articles plus tobacco companies with at least one article				
CNTC	China	2540	21755	8.56
JTI	Japan	1884	42559	22.59
PM	International	1832	41343	22.57
RJR	USA	696	13158	18.91
BAT	UK	565	8897	15.75
Chinese Academy of Science	China	253	5575	22.04
University of Tokyo	Japan	164	5591	34.09
Yunnan University	China	113	780	6.90
University of Science and Technology of China	China	108	1289	11.94
Zhejiang University	China	97	1363	14.05
Chinese Academy of Agricultural Sciences	China	93	578	6.22
Henan Agricultural University	China	90	501	5.57
Kyoto University	Japan	79	2227	28.19
Yunnan Minzu University	China	79	375	4.75
Zhengzhou University of Light Industry	China	77	350	4.55
Imperial Brands	UK	76	691	9.09
Kunming University of Science and Technology	China	69	384	5.57
Virginia Commonwealth University	USA	64	1712	26.75
Zhengzhou University	China	64	545	8.52
Sun Yat-sen University	China	59	364	6.17
Henan University of Technology	China	52	703	13.52
Sichuan University	China	51	396	7.76
Tobacco Manufacturers Association (TMA)	UK	6	4	0.67
B: S_Funder_: List threshold at least 50 articles plus tobacco companies with at least one article				
CNTC	China	1179	8573	7.27
PM	International	279	4001	14.34
BAT	UK	131	2250	17.18
Chinese Academy of Agricultural Sciences	China	130	900	6.92
Yunnan University	China	128	1513	11.82
Chinese Academy of Science	China	124	2081	16.78
Henan Agricultural University	China	116	858	7.40
Southwest University	China	106	1325	12.50
Guizhou University	China	76	367	4.83
JTI	Japan	75	1308	17.44
University of Science and Technology of China	China	68	889	13.07
Virginia Commonwealth University	USA	66	1567	23.74
North Carolina State University	USA	64	824	12.88
Zhejiang University	China	60	680	11.33
Hunan Agricultural University	China	52	287	5.52
RJR	USA	26	419	16.12
Imperial Brands	UK	14	148	10.57
Swedish Match	Sweden	9	120	13.33
Souza Cruz	Brazil	1	48	48
C: S_Title_: List threshold at least 20 articles plus tobacco companies with at least one article				
University of California. San Francisco	USA	238	7795	32.75
University of London	UK	93	1800	19.35
University of Bath	UK	61	1557	25.52
University of Sydney	Australia	50	992	19.84
Harvard University	USA	36	1244	34.56
CNTC	China	29	70	2.41
Johns Hopkins University	USA	26	393	15.12
University of Edinburgh	UK	26	706	27.15
University of York	UK	24	233	9.71
Mayo Clinic	USA	24	770	32.08
Simon Fraser University	Canada	22	160	7.27
PM	International	16	12	0.75
BAT	UK	8	33	4.13
RJR	USA	2	13	6.50
JTI	Japan	1	0	0

#### S_Affiliation_

Among the 8077 articles, the government-owned *China National Tobacco Corporation* (CNTC, China) led the ranking with a contribution to 2540 articles (31.45%). *Japan Tobacco Inc* (JTI, Japan) follows with n = 1884 (23.33%), *Philip Morris* (PM, USA and Switzerland) with n = 1832 (22.68%), *R. J. Reynolds Tobacco Company* (RJR, USA) with 694 (8.59%), and *British American Tobacco* (BAT, UK) with n = 565 (7%).

#### S_Funder_

Among the 3186 tobacco industry-funded articles, CNTC sponsored 1179 articles (37%), followed by PM with 279 (8.76%), BAT with 131 (4.11%), JTI with 75 (2.35%), RJR with 26 (0.82%), and *Imperial Brands* (IB, UK) with 14 articles (0.44%).

#### S_Title_

Among the 2204 articles about the tobacco industry, only a few were with a contribution by tobacco companies: CNTC (n = 29), PM (n = 16), BAT (n = 8), RJR (n = 2), and JTI (n = 1). Here dominated universities from the USA, UK, and Australia.

### Collaborations of the leading tobacco companies and institutions

**Figure 3.**
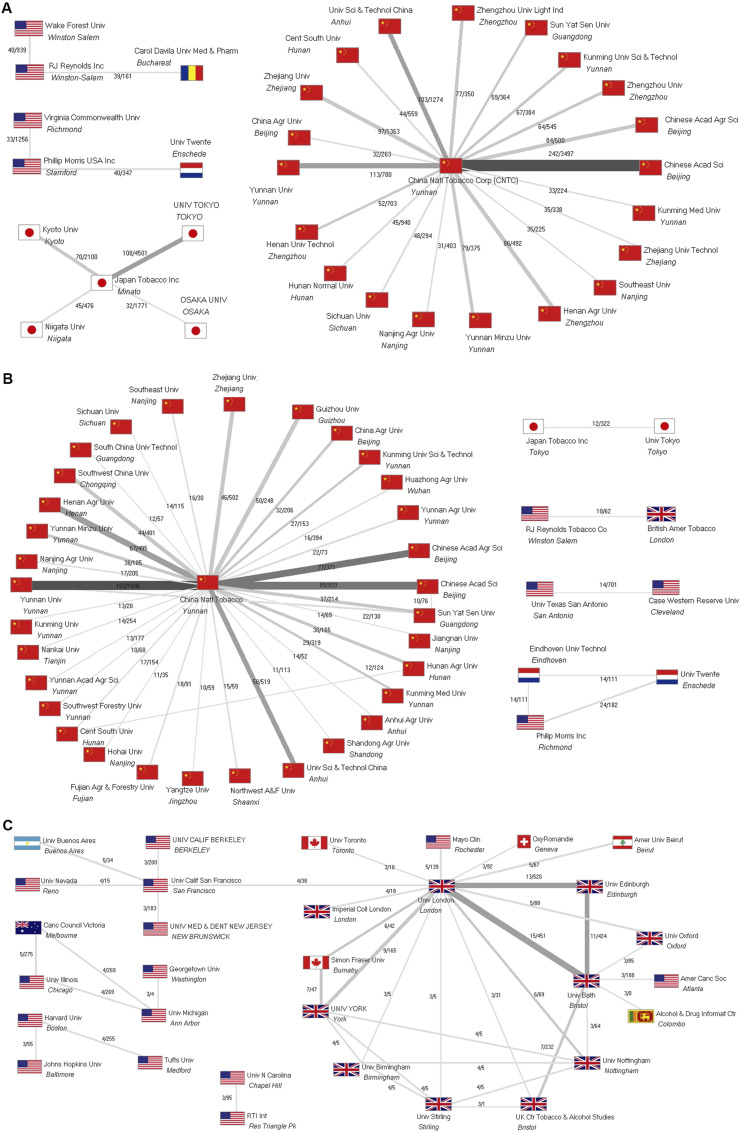
Collaboration networks of the tobacco companies and institutions. Numbers: number of common articles/number of citations. A: Publications from authors affiliated with the tobacco industry. Threshold for display: 30 collaboration articles. B: Publications funded by the tobacco industry. Threshold for display: 10 collaboration articles. C: Publications about the tobacco industry. Threshold for display: 3 collaboration articles.

Figure 3 illustrates the collaboration networks of the respective tobacco companies and institutions in the three search areas.

#### S_Affiliation_

CNTC collaborated predominantly with 18 Chinese universities, the *Chinese Academy of Sciences* (CAS), and the *Chinese Academy of Agricultural Sciences* (CAAS). The Japanese tobacco company JTI collaborated mainly with four Japanese universities. Most frequently, RJR collaborated with one US and one Romanian university, and PM with one US and one Dutch university (Figure 3(A)).

#### S_Funder_

CNTC collaborated mainly with 29 Chinese universities and three academies, CAS, CAAS, and the *Yunnan Academy of Agricultural Sciences*. A few networks among themselves between Chinese universities are visible. Additionally, four small networks collaborated on at least ten articles, namely JTI with the *University of Tokyo*, RJR with BAT, PM with two Dutch universities, and two US universities (Figure 3(B)).

#### S_Title_

The institutional collaboration networks concerning articles about the tobacco industry (display threshold of at least three collaboration articles) showed no participation of any tobacco company (Figure 3(C)). Two collaborations were found between BAT and PM, the only collaborations of tobacco companies among themselves. Most collaborating institutions were universities from the USA and the UK, besides a Swiss association against smoking (*OxyRomandie*),^18^ an independent non-profit research institute (*RTI International*, USA),^19^ a non-profit cancer charity organization (*Cancer Council Victoria*, Australia),^20^ an independent organization against drugs (*Alcohol & Drug Information Centre*, Sri Lanka),^21^ a network of 13 universities focused on spreading information on Tobacco and alcohol (*UK Centre for Tobacco and Alcohol Studies*),^22^ and the *American Cancer Society*.^23^

### Most publishing countries and international collaborations

Table 3 lists the most publishing countries, and Table 4 international collaborations in the three search areas. Supplement Figure S1 in the supplementary files shows the collaborations on articles at the country level.

**Table 3.** Most publishing countries (threshold 30 articles at search areas S_Affiliation_ and S_Funder_, 10 articles at search area S_Title_ sorted by the total number of articles with the declaration of the share of collaboration articles.

Country	Articles	Collaboration Articles	Collaboration Articles (%)
A: S_Affiliation_			
China	2628	257	9.8
USA	2403	641	26.7
Japan	2064	199	9.6
Switzerland	718	448	62.4
UK	671	320	47.7
Germany	334	318	95.2
France	90	66	73.3
Canada	88	63	71.6
Singapore	84	83	98.8
Italy	84	39	81.3
Netherlands	83	81	97.6
Belgium	70	68	97.1
Sweden	66	39	59.1
Romania	46	46	100
Australia	37	36	97.3
Spain	35	35	100
Austria	35	33	94.3
Brazil	32	17	53.1
B: S_Funder_			
China	1662	225	13.5
USA	945	376	39.8
Switzerland	266	179	67.3
UK	258	158	61.2
Germany	163	129	79.1
Japan	159	41	25.8
Canada	70	56	80
Italy	58	36	62.1
Netherlands	49	49	100
France	36	30	83.3
Sweden	35	22	62.9
Denmark	33	18	54.5
Spain	32	31	96.9
C: S_Title_			
USA	825	115	13.9
UK	276	82	29.7
Australia	123	33	26.8
China	107	19	17.8
Canada	97	45	46.4
India	38	9	23.7
Germany	31	11	35.5
Brazil	27	11	40.7
Switzerland	26	12	46.2
New Zealand	26	7	26.9
Spain	22	7	31.8
Japan	19	5	26.3
Netherlands	14	6	42.9
Thailand	13	8	61.5
Finland	13	5	38.5
Mexico	13	5	38.5
Belgium	10	8	80

**Table 4.** Bilateral collaborations at country level sorted by the number of collaboration articles in the three search areas S_Affiliation_ (threshold 20 articles), S_Funder_ (threshold 15 articles), and S_Title_ (threshold 5 articles).

Collaboration Countries	Collaboration Articles	Collaboration Countries	Collaboration Articles
A: S_Affiliation_			
USA/Switzerland	157	Switzerland/Italy	24
Switzerland/Germany	152	UK/France	23
USA/Germany	132	Singapore/Germany	22
USA/UK	131	USA/Italy	22
USA/China	111	USA/Sweden	22
USA/Japan	111	UK/Austria	21
UK/Germany	87	Switzerland/France	21
UK/Switzerland	69	USA/France	21
Switzerland/Singapore	64	USA/Belgium	20
Switzerland/Netherlands	57	UK/Canada	20
Germany/Belgium	43	UK/China	20
USA/Romania	42	UK/Italy	20
Switzerland/Japan	40	UK/Japan	20
USA/Canada	37	USA/Netherlands	20
Switzerland/Belgium	24	UK/Sweden	20
B: S_Funder_			
USA/China	115	USA/Japan	22
USA/UK	72	UK/Switzerland	20
Switzerland/Germany	64	UK/Canada	19
USA/Germany	60	USA/Italy	19
USA/Switzerland	58	Switzerland/Singapore	19
USA/Canada	31	USA/Spain	17
Switzerland/Netherlands	30	UK/Italy	16
UK/Germany	24	UK/China	15
Pakistan/China	22	USA/Netherlands	15
C: S_Title_			
USA/UK	22	USA/Brazil	7
USA/Canada	21	UK/Lebanon	6
UK/Canada	18	UK/Switzerland	6
USA/Australia	13	USA/Argentina	5
UK/Australia	9	USA/Germany	5
USA/China	8		

#### S_Affiliation_

The six most publishing countries were China, the USA, Japan, and, followed by a considerable gap, Switzerland, the UK, and Germany (Table 3A). These countries were collaborating first of all among themselves, especially the USA with Switzerland, Germany, the UK, China, or Japan, and Switzerland with Germany, to name the leading six bilateral collaborations (Table 4A, Supplement Figure S1(A)).

#### S_Funder_

The six most publishing countries were China and the USA, followed by a bigger gap by Switzerland, the UK, Germany, and Japan (rank six in place of three) (Table 3B). The most bilateral networking occurred again between the USA and China, the UK, Germany, Switzerland, and Canada. Another identified network among the first six was between Switzerland and Germany on rank three (Table 4B, Supplement Figure S1(B)).

#### S_Title_

The most publishing country was the USA, followed by a bigger gap by the UK, Australia, China, Canada, and India, to name the leading six (Table 3C). Germany followed on rank seven, Switzerland nine, and Japan on rank 12. Most collaborations occurred between the USA and the UK, Canada, Australia, and China, and, additionally, between the UK and Canada and Australia, respectively (Table 4C, Figure S1(C)).

### Research funding

#### S_Affiliation_

Of the overall 8077 articles, 2895 articles (35.84%) were funded and received in sum 6378 grants (g), of them g = 1596 (25.02%) from the tobacco industry, including g = 981 (61.47%) from CNTC, g = 324 (20.3%) from PM, g = 138 (8.65%) from BAT, g = 73 from JTI (4.57%), g = 28 (1.75%) from RJR, and g = 27 (1.69%) from IB, to name the six most funding tobacco companies.

#### S_Funder_

Among the 3186 tobacco industry-funded articles, from 9136 grants, g = 3380 (37%) could be assigned to the tobacco industry. Of these, g = 1938 (57.34%) were awarded by CNTC, followed by PM with g = 914 (27.04%), BAT with g = 178 (5.27%), JTI with g = 134 (3.96%), RJR with g = 96 (2.84%), IB with g = 46 (1.36%), and Swedish Match with g = 25 (0.74%), to name the companies with at least two digits quantity of grants they awarded.

#### S_Title_

In 2204 articles, only 12 tobacco industry assignable grants from overall g = 1293 were found, meaning less than 1%, eight from CNTC, and each one from PM, BAT, Tabacalera SLU (Cuba), and Tabacuba (Cuba).

### Most publishing journals

Table 5 lists the 15 most publishing journals sorted by the number of articles in the three analyzed search areas, including information about the respective journal category (subject area) and the *Journal Impact Factors* (JIFs) from the database of the *Journal Citation Reports™* from Clarivate (accessed on 23 February 2023).

**Table 5.** The 15 most publishing journals sorted by the number of articles of the three search areas (S_Affiliation_, S_Funder_, S_Title_) analyzed. In brackets are the ournal Impact Factors From Clarivates’ Journal Citation Reports™ (accessed on 23 February 2023). n/a = not available (not indexed in Journal Citation Reports database).

Journal (Impact Factor)	Journal Category (Subject Area)	Articles	Citations	Citation Rate
A: S_Affiliation_				
Agricultural and Biological Chemistry (n/a)	Bioscience, Biotechnology, Agrochemistry	252	3311	13.14
Abstracts of Papers of the American Chemical Society (n/a)	Chemistry	179	3	0.02
Regulatory Toxicology and Pharmacology (3.598)	Toxicology, Pharmacology, Legal Medicine	168	3532	21.02
Toxicology Letters (4.271)	Toxicology	154	394	2.56
Journal of Chromatography A (4.601)	Chemistry, Biochemical Research Methods	89	1937	21.76
Inhalation Txicology (3.011)	Toxicology	89	1509	16.96
Food and Chemical Toxicology (5.572)	Toxicology, Food Science & Technology	88	3489	39.65
Journal of Agricultural and Food Chemistry (5.895)	Chemistry, Agriculture, Food Science & Technology	76	1795	23.62
Heterocycles (0.689)	Chemistry	66	702	10.64
Journal of Analytical and Applied Pyrolysis (6.437)	Chemistry, Energy & Fuels, Chemical Engineering	65	2596	39.94
Phytochemistry (4.004)	Biochemistry & Molecular Biology, Plant Sciences	64	1341	20.95
Contributions to Tobacco Research International (n/a)	Agricultural and Biological Sciences	62	552	8.90
Mutation Research (2.107)	Toxicology, Genetics & Heredity	62	1291	20.82
Journal of Chromatographic Science (1.555)	Chemistry, Biochemical Research Methods	58	1001	17.26
Scientific Reports (4.997)	Multidisciplinary Sciences	55	565	10.27
B: S_Funder_				
Regulatory Toxicology and Pharmacology (3.598)	Toxicology, Pharmacology, Legal Medicine	81	1227	15.15
Scientific Reports (4.997)	Multidisciplinary Sciences	50	625	12.50
Nicotine & Tobacco Research (5.825)	Public, Environmental & Occupational Health, Substance Abuse	40	844	21.10
Chemistry of Natural Compounds (0.830)	Chemistry	33	95	2.88
International Journal of Systematic and Evolutionary Microbiology (2.689)	Microbiology	27	188	0.14
Asian Journal of Chemistry (0.355)	Chemistry	25	61	0.41
Journal of Separation Science (3.614)	Chemistry	25	194	0.13
Toxicology Letters (4.271)	Toxicology	23	141	0.16
Toxicology In Vitro (3.685)	Toxicology	23	501	0.05
Inhalation Toxicology (3.011)	Toxicology	23	478	0.05
Plant Disease (4.614)	Plant Sciences	22	78	0.28
Heterocycles (0.689)	Chemistry	22	181	0.12
Food and Chemical Toxicology (5.572)	Toxicology, Food Science & Technology	20	443	0.05
Journal of Agricultural and Food Chemistry (5.895)	Chemistry, Agriculture, Food Science & Technology	20	316	0.06
Journal of Aerosol Science (4.586)	Engineering, Meteorology & Atmospheric Sciences, Environmental Sciences	20	366	0.05
C: S_Title_				
Tobacco Control (6.953)	Public, Environmental & Occupational Health, Substance Abuse	406	8581	21.14
The BMJ (96.216)	General & Internal Medicine	176	629	3.57
Lancet (202.731)	General & Internal Medicine	104	853	8.20
American Journal of Public Health (11.576)	Public, Environmental & Occupational Health	72	2715	37.71
Tobacco Induced Diseases (5.163)	Public, Environmental & Occupational Health, Substance Abuse	68	7	0.10
JAMA-Journal of the American Medical Association (157.375)	General & Internal Medicine	35	1104	31.54
Nicotine & Tobacco Research (5.825)	Public, Environmental & Occupational Health, Substance Abuse	34	603	17.74
Addiction (7.256)	Psychiatry, Substance Abuse	29	591	0.05
PLOS Medicine (11.613)	General & Internal Medicine	17	698	41.06
European Journal of Public Health (4.424)	Public, Environmental & Occupational Health	17	175	0.10
Canadian Medical Association Journal (16.876)	General & Internal Medicine	16	20	0.80
Global Public Health (3.356)	Public, Environmental & Occupational Health	16	157	0.10
American Journal of Agricultural Economics (3.757)	Economics	14	99	0.14
Fortune (0.285)	Business	12	2	0.17
Medical Journal of Australia (12.776)	General & Internal Medicine	12	12	1

#### S_Affiliation_

Of the 15 most publishing journals, the first 14 were widely in the fields of biosciences, chemistry, toxicology, engineering, and plant or agricultural sciences. A multidisciplinary journal ranked 15. Disciplines of medicine were not listed, except legal medicine in one case. The JIFs were all below seven, and the top two journals were not indexed in the *Journal Citation Reports™* database. Of note, the 12^th^-ranked German Journal, *Contributions to Tobacco Research International* (original German title: *Beiträge zur Tabakforschung,* currently: *Contributions to Tobacco & Nicotine Research*) was originally published by the German Association of Cigarette Industries (German: VdC, *Verband der Cigarettenindustrie*).^24^

#### S_Funder_

Again, most of the journals listed were in the categories of chemistry, toxicology, engineering respective technology, and plant or agricultural sciences. In second place was a multidisciplinary journal, and in third was a journal in the fields of public, environmental and occupational health and substance abuse, respectively. The JIFs were all below six.

#### S_Title_

A different picture emerged here. All of the journals listed belonged to the medical categories, except one in economics (rank 13) and one in business (rank 14). Moreover, three markedly high-impact journals were found with JIFs of approximately 100 and above. Another four journals had JIFs above 10.

## Discussion

The tobacco industry is increasingly involved in research. Thus, policymakers, consumers, academics, and, not least, publishers should all remain cautious, given the industry’s documented history of attempting to influence science for its gain at the expense of public health.

Therefore, we analyzed the publication behavior and topics of the tobacco industry in scientific publications using the WoS database. We sectioned this bibliometric study into three investigation areas: (1) the investigation of publications of authors affiliated with the tobacco industry, (2) publications funded by them, and (3) publications about the tobacco industry to identify differences compared to investigation areas (1) and 2).

First, we looked at the total number of articles, their citation numbers, the resulting citation rates of each search area, and their development over time. The first identified article about the tobacco industry was published in 1900 in JAMA.^17^ We found only 32 articles until 1968 inclusive. This trend is similar to the development of publications of tobacco industry-affiliated authors (n = 9). Until this time, it would seem that the interest in scientific publications on the part of both the tobacco industry and the scientific community in studies about the tobacco industry was limited. We found no publications in the WoS database as a direct response to findings published in 1939, 1950, or the 1960s concerning health effects like lung cancer or heart disease^1–3^ by authors affiliated with the tobacco industry, nor to the 1964 report of health hazards by smoking by the US Surgeon General.^25^ Our study confirms insofar that the tobacco industry’s strategies were more likely the reputational damage of science through PR campaigns.^5^ The 1972 report of the US Surgeon General was the first report on the health consequences of smoking, which included the health hazards of passive smoking.^26^ The tobacco industry answered with scientific, public, and political disinformation campaigns and sponsored research to trivialize the health hazard of passive smoking.^27^ Since 1972, the relative share of publications in which tobacco industry-affiliated authors were involved increased rapidly, indicating an increase in research interest on the side of the tobacco industry. The first studies we found by tobacco industry-affiliated authors that addressed the chemical or biological effects of tobacco ingredients or cigarette smoke condensate were published in 1973 without explicit mention of the health hazards of smoking.^28-31^ According to the WoS research areas, studies associated with or funded by the tobacco industry were largely unrelated to health issues. Not surprisingly, there were few studies whose authors were affiliated with or were funded by the tobacco industry and that addressed the health effects of smoking. By contrast, many articles about the tobacco industry were published in journals dealing with health topics. That was also underlined by the subject areas of the most-publishing journals. Articles by tobacco industry-affiliated authors and tobacco industry-funded articles were mainly published in journals dealing with chemistry, toxicology, or agriculture, and articles about the tobacco industry, by contrast, mainly in journals with health-related topics. Nevertheless, the funding of medical research by tobacco companies is ubiquitous, even if it is only that, eg, health-related foundations receive money from them.^32^

As other studies have already shown, institutional scientific collaborations have generally fanned out into broad networks.^33,34^ That is also true for articles about the tobacco industry. The situation is quite different for studies in which the tobacco industry was involved, either with affiliated authors or through funding. The networks of the Chinese governmental tobacco company CNTC with many Chinese academic institutions are remarkable. Besides its research capacity, CNTC benefits from Chinese universities to further its research agenda, primarily in agriculture, production, and business management, but, increasingly, also in the so-called ‘less harmful, low-tar’ strategy.^35^ Networks of Western universities with tobacco companies could only be detected on a much lower level, as they presumably are wary of relationships with the tobacco industry due to their reputation.^35^ In line with these findings, for China, besides Japan, collaborations at the country level are below average. That also indicates that research involving tobacco companies (here: CNTC and JTI, respectively) is more likely to be interest-driven. Possibly, international collaborations would be more of a hindrance than a help here, also because the tobacco industry may have greater control over narratives and communities within their “hometown”.

CNTC is the largest tobacco company in the world.^36^ We have found that CNTC is also the most research-supporting tobacco company by far, both by CNTC-affiliated authors and funding. The other ‘Big Tobacco’, Philip Morris, British American Tobacco, Japan Tobacco, Imperial Brands, but even RJ Reynolds, follow at some distance in research support, at least as far as non-disguised funding is concerned. By its very nature, the tobacco industry is virtually uninvolved in studies about themselves. However, they have a long history of establishing third-party organizations to fund research and scientists. That leads naturally to conflicts of interest.^37^ It has become known that the tobacco industry uses scientists and research projects for its purposes and continues to systematically stir up controversies about the health hazards of smoking and scientific evidence.^38^ Notably, about one-third of publications with tobacco industry-affiliated authors received grants from the tobacco industry. Not surprisingly, articles about the tobacco industry were almost not funded by the industry.

Studies on internal tobacco industry documents revealed post hoc protocol changes and biased experimental set-ups in scientific publications linked to tobacco companies.^39,40^ Tobacco industry research did not meet standards for clinical research.^41^ Publishing tobacco industry-affiliated or funded research in scientific journals is controversially discussed.^42^ Several journals and academic institutions decided to stop publishing tobacco industry-supported research.^43,44^ Unfortunately, even leading medical journals have no tobacco policies that prohibit studies funded by the tobacco industry.^45^ The question arises whether rather medical journals fear to publish studies with results favorable to the tobacco industry or are non-medical journals regarding tobacco policies by far less restrictive. Future studies should analyze the publication policies of scientific journals and publishers. On the other hand, the tobacco industry publishes in non-peer-reviewed journals that suggest meeting scientific standards. Funding and disseminating non-peer-reviewed research is a longstanding strategy.^44^ That could also be true for medical journals and research, leading to lower entries in the WoS database as WoS does not list non-peer-reviewed journals. Journals in the fields where the tobacco industry provides extraordinary support (eg, chemistry, toxicology, pharmacology, or agriculture) would do well to be skeptical, not least because the tobacco industry is interested in normalization of their presence within science and academia, even if it’s not dealing with tobacco.^37^ The presented findings of this study enable the interpretation of trends, focal points, and linkages in tobacco industry-related research and their significance for future research projects.

All bibliometric studies have limitations depending on the used database and search strategies. The applied core collection of WoS has strict indexing requirements for journals. For example, it only contains journals that practice a standard peer-review process, so articles published in non-indexed journals are not included in the analysis. Journals that do not meet the requirements of WoS are not listed. The English bias of WoS favors English literature, so not all related publications in other languages could be included in the analysis database. WoS cannot filter out incorrect or self-citations. That can distort figures or ratios for all citation parameters. Publications before 1900 were not included as this is the lower limit of the evaluation period of WoS. In addition, WoS began just in August 2008 to collect data systematically on funding agencies.^15^ Therefore, the analyses of articles funded by the tobacco industry could only be carried out from this year onward. The conclusions about the funding activity of the tobacco industry must be assessed in this context. It must also be said that not all tobacco companies could necessarily be found by the applied search strings. That is also a limitation inherent in most bibliometric analyses. To minimize this incompleteness, we included general company name affixes like, eg, tobacco industry or company with the variations of notation in addition to the names of the bigger tobacco companies. Not all authors’ affiliations with the tobacco industry could be identified, as they are sometimes hidden or not declared, or the authors are affiliated with third parties with close ties to the industry. Authors’ conflicts of interest with the tobacco industry are not always declared in academic publications.^46^ Such is true for funding activities, in particular, if funding occurred indirectly by third parties. This limitation led to a decrease in data. As the representativeness of the generated database is mandatory, a reduction in the number of entries is acceptable.

## Conclusion

Tobacco companies have promoted their business interests through PR campaigns and lobbying in science and academia, including by damaging the reputation of science, conducting their “own” research, or providing specific financial support. Studies by tobacco industry-affiliated authors or sponsored by the tobacco industry are less likely to be published in health-related peer-reviewed journals but more in chemistry, toxicology, pharmacology, or agricultural sciences. The Chinese governmental CNTC is the most research-supporting tobacco company, followed by the other ‘Big Tobacco’ companies. Certainly, conflicts of interest occur time and again. The scientific community, researchers, academic institutions, journals, and health-related science, in particular, should be skeptical about tobacco industry-supported research. The best way to control corporate influence on science is possibly structural changes in funding science, eg, transparent and independent funding processes where payments from the industry are independently administered.^47^ Education programs like, eg, the Education Against Tobacco (EAT) prevention program^48^ are helpful and necessary to reduce smoking prevalence and to raise awareness of the influence of the tobacco industry on science, policy, and public perception.

## Supplemental Material

Supplemental Material - Activity of the Tobacco Industry in Research and Scientific Literature Supplemental Material for Activity of the Tobacco Industry in Research and Scientific Literature by Markus Braun, Doris Klingelhöfer, Dörthe Brüggmann and David A. Groneberg in Tobacco Use Insights.

## Data Availability

Bibliometric data are owned by and were obtained from the Web of Science database. The authors are not allowed to share the data publicly or privately. Any researcher with access to the Web of Science database can obtain the data using the methods described in the paper.
