# Supplementary material for: Activity of the Tobacco Industry in Research and Scientific Literature
Source: Tob Use Insights. 2024 Aug 19;17:1179173X241271566. doi: 10.1177/1179173X241271566 (PMC11334150; doi:10.1177/1179173X241271566)
Supplement: Supplemental Material - Activity of the Tobacco Industry in Research and Scientific Literature [file sj-pdf-1-tui-10.1177_1179173X241271566.pdf]

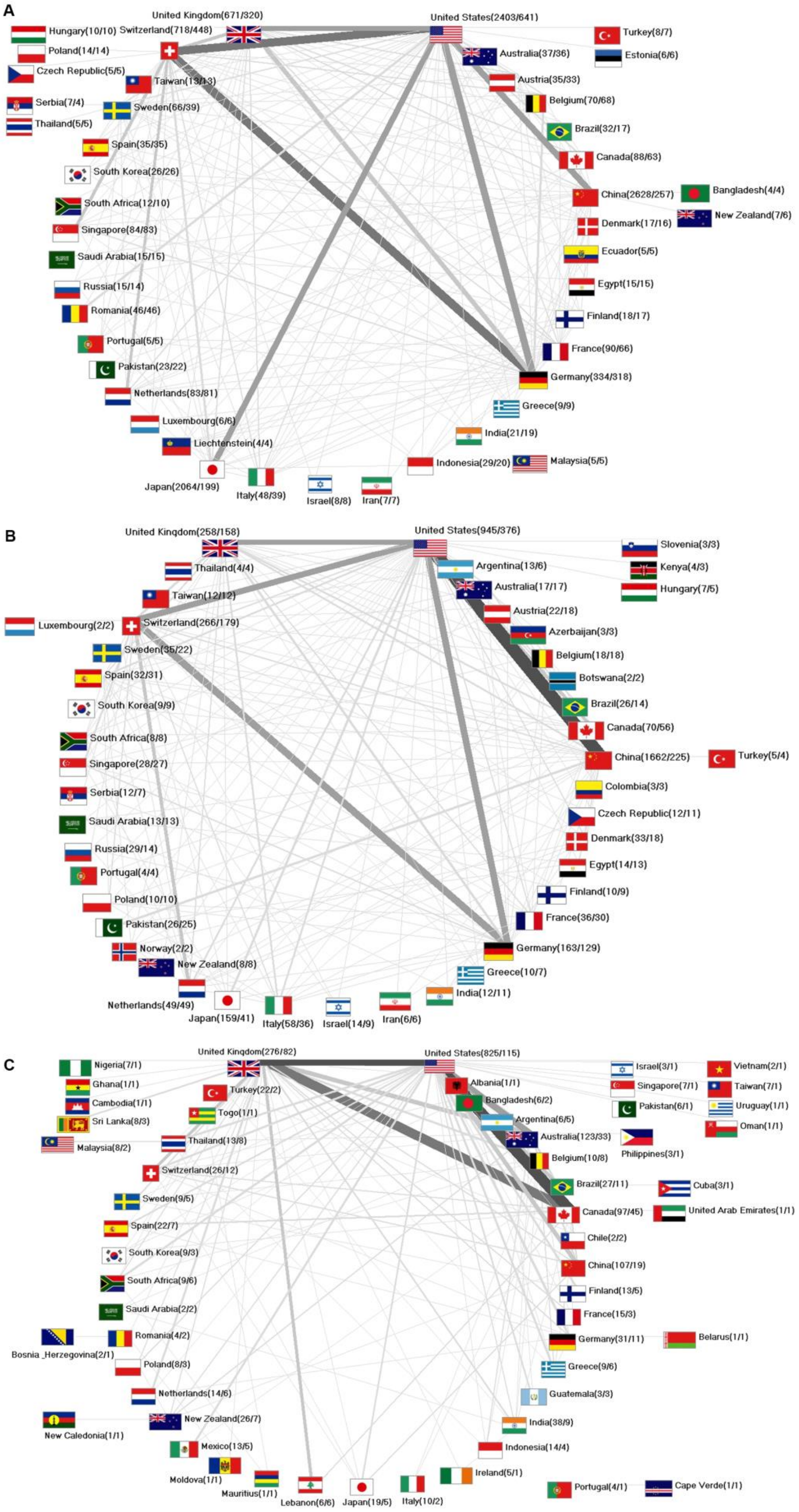

Figure S1: International networks. Numbers in brackets (number of total articles/number of collaboration articles). A: Publications from authors affiliated with the tobacco industry. Threshold for display: 3 collaboration articles. B: Publications funded by the tobacco industry. Threshold for display: 2 collaboration articles. C: Publications about the tobacco industry. Threshold for display: 1 collaboration article.

Table S1: Ten most cited articles sorted by number of citations in the three search areas ($S_{Affiliation}$, S_{Funder}, S_{Title}) analyzed.

Authors	Country	Year	Citations	Title	Journal
A: $S_{Affiliation}$					
Hiei, Y et al.	Japan	1994	2470	Efficient Transformation of Rice (Oryza-Sativa L) mediated by Agrobacterium and Sequence-Analysis of the Boudaries of the T-DNA	Plant Journal
Hu, M et al.	USA, China	2006	1397	Gold nanostructures: engineering their plasmonic properties for biomedical applications	Chemical Society Reviews
Guex, N et al.	Switzerland	2009	1047	Automated comparative protein structure modeling with SWISS-MODEL and Swiss-PdbViewer: A historical perspective	Electrophoresis
Stanke, M et al.	Germany, USA	2006	808	Augustus: ab initio prediction of alternative transcripts	Nuclear Acid Research
Seeman, JI	USA	1983	797	Effect of Conformational Change in Reactivity in Organic-Chemistry - Evaluations, Applications, and Extensions of Curtin-Hammett Winstein-Holness Kinetics	Chemical Reviews
Sharma, RK et al.	USA	2004	648	Characterization of chars from pyrolysis of lignin	Fuel
Deevi, SC and Sikka, VK	USA	1996	587	Nickel and iron aluminides: An overview on properties, processing, and applications	Intermetallics
Ishida, Y et al.	Japan	1996	571	High efficiency transformation of maize (Zea mays L) mediated by Agrobacterium tumefaciens	Nature Biotechnology
Utada, AS et al.	USA	2007	552	Dripping to jetting transitions in coflowing liquid streams	Physical Review Letters
Link, DR et al.	USA	2006	528	Electric control of droplets in microfluidic devices	Angewandte Chemie International
B: S_{Funder}					
Park, EJ et al.	USA	2010	1123	Dietary and Genetic Obesity Promote Liver Inflammation and Tumorigenesis by Enhancing IL-6 and TNF Expression	Cell
Kilgore, M et al.	USA	2010	472	Inhibitors of Class 1 Histone Deacetylases Reverse Contextual Memory Deficits in a Mouse Model of Alzheimer's Disease	Neuropsychopharmacology
Mishra, SI et al.	USA, Canada	2012	455	Exercise interventions on health-related quality of life for cancer survivors	Cochrane Database of Systematic Reviews
Foltz, DR et al.	USA	2009	445	Centromere-Specific Assembly of CENP-A Nucleosomes Is Mediated by HJURP	Cell
Reddy, TE et al.	USA	2009	346	Genomic determination of the glucocorticoid response reveals unexpected mechanisms of gene regulation	Genome Research

Dawkins, L et al.	UK	2013	341	'Vaping' profiles and preferences: an online survey of electronic cigarette users	Addiction
Epsztejn-Litman, S et al.	Israel, Japan, Belgium	2008	319	De novo DNA methylation promoted by G9a prevents reprogramming of embryonically silenced genes	Nature Structural & Molecular Biology
Csiszar, A et al.	USA	2008	316	Inflammation and endothelial dysfunction during aging: role of NF-kappa B	Journal of Applied Physiology
Sierro, N et al.	Switzerland	2014	286	The tobacco genome sequence and its comparison with those of tomato and potato	Nature Communications
Bromberg, L et al.	USA	2012	280	Chromium(III) Terephthalate Metal Organic Framework (MIL-101): HF-Free Synthesis, Structure, Polyoxometalate Composites, and Catalytic Properties	Chemistry of Materials
C: S_{Title}					
Pierce, JP et al.	USA	1998	412	Tobacco industry promotion cigarettes and adolescent smoking	JAMA - Journal of the American Medical Association
Ling, PM and Glantz, SA	USA	2002	357	Why and how the tobacco industry sells cigarettes to young adults: Evidence from industry documents	American Journal of Public Health
Malone, RE and Balbach, ED	USA	2000	250	Tobacco industry documents: treasure trove or quagmire?	Tobacco Control
Wakefield, M et al.	Australia, USA	2002	218	The cigarette pack as image: new evidence from tobacco industry documents	Tobacco Control
Chapman, S and Freeman, B	Australia	2008	193	Markers of the denormalisation of smoking and the tobacco industry	Tobacco Control
Chaloupka, FJ et al.	USA	2002	185	Tax, price and cigarette smoking: evidence from the tobacco documents and implications for tobacco company marketing strategies	Tobacco Control
Bero, LA	USA	2003	184	Implications of the tobacco industry documents for public health and policy	Annual Review of Public Health
Palazzo, G et al.	Switzerland	2005	178	CSR business as usual? The case of the tobacco industry	Journal of Business Ethics
Cataldo, JK et al.	USA	2010	175	Cigarette Smoking is a Risk Factor for Alzheimer's Disease: An Analysis Controlling for Tobacco Industry Affiliation	Journal of Alzheimer's Disease
Dearlove, JV et al.	USA	2002	168	Tobacco industry manipulation of the hospitality industry to maintain smoking in public places	Tobacco Control

Table S2: All assigned Web of Science (WoS) research areas with at least one article (threshold) sorted by the number of articles of the three search areas ($S_{Affiliation}$, S_{Funder}, S_{Title}) analyzed with percentage data of the respective categories.

Rank	WoS research area	Number of articles	Percentage	Citations	Citation rate
A: $S_{Affiliation}$					
1	Chemistry	2241	18.73	35258	15.73
2	Toxicology	1128	9.43	19520	17.30
3	Biochemistry & Molecular Biology	959	8.02	22161	23.11
4	Pharmacology & Pharmacy	872	7.29	12953	14.85
5	Engineering	599	5.01	9206	15.37
6	Plant Sciences	465	3.89	10892	23.42
7	Food Science & Technology	437	3.65	8616	19.72
8	Agriculture	432	3.61	5015	11.61
9	Materials Science	392	3.28	9051	23.09
10	Genetics & Heredity	251	2.10	4793	19.10
11	Environmental Sciences & Ecology	241	2.01	3502	14.53
12	Biotechnology & Applied Microbiology	235	1.96	4432	18.86
13	Science & Technology - Other Topics	198	1.66	3937	19.88
14	Physics	198	1.66	5317	26.85
15	Energy & Fuels	160	1.34	5538	34.61
16	Spectroscopy	151	1.26	1798	11.91
17	Computer Science	149	1.25	425	2.85
18	Cell Biology	145	1.21	4611	31.80
19	Microbiology	130	1.09	1576	12.12
20	Entomology	118	0.99	1301	11.03
21	Public, Environmental & Occupational Health	116	0.97	1455	12.54
22	Polymer Science	112	0.94	1670	14.91
23	Business & Economics	111	0.93	445	4.01
24	Neurosciences & Neurology	110	0.92	3238	29.44
25	Oncology	110	0.92	2313	21.03
26	Physiology	91	0.76	1379	15.15
27	Instruments & Instrumentation	84	0.70	1185	14.11
28	Biophysics	78	0.65	2676	34.31
29	Research & Experimental Medicine	77	0.64	2231	28.97
30	Cardiovascular System & Cardiology	67	0.56	1148	17.13
31	Endocrinology & Metabolism	66	0.55	1006	15.24
32	Metallurgy & Metallurgical Engineering	65	0.54	2828	43.51
33	Immunology	65	0.54	1988	30.58
34	Mathematics	59	0.49	1233	20.90
35	Thermodynamics	57	0.48	486	8.53
36	Psychology	57	0.48	1124	19.72
37	Meteorology & Atmospheric Sciences	57	0.48	605	10.61
38	General & Internal Medicine	55	0.46	241	4.38
39	Substance Abuse	54	0.45	916	16.96
40	Respiratory System	45	0.38	548	12.18
41	Pathology	43	0.36	857	19.93
42	Veterinary Sciences	42	0.35	381	9.07
43	Electrochemistry	41	0.34	516	12.59
44	Nutrition & Dietetics	40	0.33	947	23.68
45	Virology	39	0.33	1792	45.95
46	Mathematical & Computational Biology	39	0.33	722	18.51
47	Automation & Control Systems	38	0.32	18	0.47
48	Psychiatry	34	0.28	914	26.88
49	Optics	33	0.28	203	6.15
50	Mechanics	33	0.28	171	5.18
51	Crystallography	32	0.27	76	2.38
52	Urology & Nephrology	30	0.25	639	21.30
53	Health Care Sciences & Services	29	0.24	82	0
54	Telecommunications	25	0.21	63	0
55	Zoology	23	0.19	343	0
56	Dermatology	21	0.18	478	0

57	Hematology	21	0.18	435	0
58	Education & Educational Research	20	0.17	14	0
59	Life Sciences & Biomedicine - Other Topics	17	0.14	52	3.06
60	Social Sciences - Other Topics	17	0.14	12	0.71
61	Operations Research & Management Science	17	0.14	129	7.59
62	Water Resources	17	0.14	228	0
63	Gastroenterology & Hepatology	13	0.11	138	0
64	Government & Law	12	0.10	2	0
65	Evolutionary Biology	12	0.10	96	0
66	Radiology, Nuclear Medicine & Medical Imaging	12	0.10	105	8.75
67	Nuclear Science & Technology	11	0.09	48	0
68	Imaging Science & Photographic Technology	11	0.09	9	0.82
69	Mathematical Methods In Social Sciences	11	0.09	313	28.45
70	Medical Laboratory Technology	11	0.09	125	0
71	Information Science & Library Science	10	0.08	5	0.50
72	Remote Sensing	9	0.08	19	0
73	Communication	9	0.08	33	0
74	Ophthalmology	9	0.08	19	0
75	Behavioral Sciences	8	0.07	148	0
76	Dentistry, Oral Surgery & Medicine	8	0.07	37	0
77	Anatomy & Morphology	7	0.06	146	0
78	Developmental Biology	7	0.06	247	0
79	Acoustics	6	0.05	14	0
80	Mycology	6	0.05	35	0
81	Integrative & Complementary Medicine	6	0.05	99	16.50
82	Allergy	6	0.05	196	0
83	Microscopy	5	0.04	106	0
84	Reproductive Biology	5	0.04	65	0
85	Obstetrics & Gynecology	5	0.04	57	0
86	Fisheries	5	0.04	109	0
87	Urban Studies	4	0.03	1	0
88	Transportation	4	0.03	2	0
89	Public Administration	4	0.03	16	0
90	Parasitology	4	0.03	338	0
91	Transplantation	4	0.03	1	0
92	Audiology & Speech-Language Pathology	4	0.03	1	0
93	Geology	3	0.03	32	0
94	Social Issues	3	0.03	3	0
95	Robotics	3	0.03	0	0
96	PublicAdministration	3	0.03	0	0
97	Otorhinolaryngology	3	0.03	55	0
98	Geriatrics & Gerontology	3	0.03	26	0
99	Orthopedics	2	0.02	69	0
100	Surgery	2	0.02	80	0
101	Rheumatology	2	0.02	49	0
102	Physical Geography	2	0.02	2	0
103	Pediatrics	2	0.02	23	0
104	Forestry	2	0.02	0	0
105	Sport Sciences	1	0.01	4	0
106	Construction & Building Technology	1	0.01	7	0
107	Anthropology	1	0.01	60	0
108	Art	1	0.01	0	0
109	TropicalMedicine	1	0.01	22	0
110	Geochemistry & Geophysics	1	0.01	4	0
111	Biomedical Social Sciences	1	0.01	2	0
112	Oceanography	1	0.01	50	0
113	Architecture	1	0.01	0	0
114	Anesthesiology	1	0.01	24	0
115	Medical Informatics	1	0.01	2	0
116	Literature	1	0.01	1	0
117	Arts & Humanities - Other Topics	1	0.01	0	0
118	Biodiversity & Conservation	1	0.01	122	0
119	Womens Studies	1	0.01	19	0

120	International Relations	1	0.01	0	0
B: S_{Funder}					
1	Chemistry	569	12.36	7001	12.30
2	Pharmacology & Pharmacy	330	7.17	5776	17.50
3	Toxicology	319	6.93	5244	16.44
4	Plant Sciences	274	5.95	2719	9.92
5	Biochemistry & Molecular Biology	246	5.34	6759	27.48
6	Agriculture	204	4.43	1237	6.06
7	Engineering	187	4.06	2295	12.27
8	Environmental Sciences & Ecology	172	3.74	2194	12.76
9	Science & Technology - Other Topics	143	3.11	4080	28.53
10	Microbiology	131	2.84	1243	9.49
11	Biotechnology & Applied Microbiology	125	2.71	2407	19.26
12	Materials Science	122	2.65	2772	22.72
13	Food Science & Technology	114	2.48	1327	11.64
14	Neurosciences & Neurology	114	2.48	2727	23.92
15	Genetics & Heredity	103	2.24	2038	19.79
16	Public, Environmental & Occupational Health	85	1.85	1416	16.66
17	Entomology	76	1.65	375	4.93
18	Cell Biology	72	1.56	4600	63.89
19	Substance Abuse	65	1.41	1564	24.06
20	Physics	63	1.37	779	12.37
21	Psychiatry	54	1.17	2425	44.91
22	Spectroscopy	47	1.02	348	7.40
23	Energy & Fuels	45	0.98	895	19.89
24	Research & Experimental Medicine	44	0.96	939	21.34
25	Biophysics	43	0.93	1502	34.93
26	Oncology	43	0.93	1795	41.74
27	Physiology	41	0.89	1220	29.76
28	Polymer Science	39	0.85	379	9.72
29	Virology	38	0.83	619	16.29
30	Meteorology & Atmospheric Sciences	38	0.83	546	14.37
31	Life Sciences & Biomedicine - Other Topics	36	0.78	507	14.08
32	Computer Science	36	0.78	157	4.36
33	Cardiovascular System & Cardiology	35	0.76	1226	35.03
34	Immunology	30	0.65	1112	37.07
35	Mathematics	25	0.54	244	0
36	Instruments & Instrumentation	23	0.50	282	12.26
37	General & Internal Medicine	23	0.50	1347	0
38	Mathematical & Computational Biology	23	0.50	456	0
39	Thermodynamics	22	0.48	142	0
40	Rheumatology	22	0.48	637	0
41	Zoology	21	0.46	121	0
42	Respiratory System	20	0.43	994	0
43	Urology & Nephrology	20	0.43	693	0
44	Endocrinology & Metabolism	19	0.41	639	0
45	Psychology	16	0.35	326	0
46	Mechanics	14	0.30	217	0
47	Radiology, Nuclear Medicine & Medical Imaging	13	0.28	206	15.85
48	Electrochemistry	12	0.26	311	0
49	Hematology	12	0.26	611	0
50	Dermatology	12	0.26	426	0
51	Nutrition & Dietetics	11	0.24	133	0
52	Pathology	11	0.24	260	0
53	Water Resources	10	0.22	104	0
54	Metallurgy & Metallurgical Engineering	9	0.20	40	0
55	Behavioral Sciences	8	0.17	247	0
56	Dentistry, Oral Surgery & Medicine	8	0.17	60	0
57	Health Care Sciences & Services	8	0.17	50	0
58	Evolutionary Biology	7	0.15	27	0
59	Gastroenterology & Hepatology	7	0.15	288	0
60	Automation & Control Systems	6	0.13	3	0
61	Geology	6	0.13	82	0

62	Ophthalmology	6	0.13	173	0
63	Business & Economics	6	0.13	12	0
64	Pediatrics	6	0.13	149	0
65	Remote Sensing	5	0.11	40	0
66	Medical Laboratory Technology	5	0.11	85	0
67	Surgery	5	0.11	196	0
68	Criminology & Penology	5	0.11	4	0
69	Social Work	5	0.11	76	0
70	Allergy	5	0.11	319	0
71	Mycology	4	0.09	14	0
72	MathematicalMethods In Social Sciences	4	0.09	38	0
73	Developmental Biology	4	0.09	101	0
74	Microscopy	4	0.09	11	0
75	Reproductive Biology	4	0.09	39	0
76	Infectious Diseases	4	0.09	196	0
77	Imaging Science & Photographic Technology	3	0.07	23	7.67
78	Physical Geography	3	0.07	17	0
79	Forestry	3	0.07	7	0
80	Telecommunications	3	0.07	1	0
81	Geochemistry & Geophysics	3	0.07	59	0
82	Geriatrics & Gerontology	3	0.07	141	0
83	Transplantation	3	0.07	75	0
84	Optics	3	0.07	48	0
85	Biodiversity & Conservation	2	0.04	85	0
86	Social Sciences - Other Topics	2	0.04	0	0
87	Parasitology	2	0.04	123	0
88	Anatomy & Morphology	2	0.04	6	0
89	Sport Sciences	2	0.04	320	0
90	Otorhinolaryngology	2	0.04	71	0
91	Marine & Freshwater Biology	2	0.04	26	0
92	Veterinary Sciences	2	0.04	4	0
93	Education & Educational Research	2	0.04	0	0
94	Crystallography	2	0.04	13	0
95	Fisheries	2	0.04	2	0
96	Geography	1	0.02	9	0
97	Public Administration	1	0.02	9	0
98	Government & Law	1	0.02	1	0
99	Integrative & ComplementaryMedicine	1	0.02	14	0
100	Operations Research & Management Science	1	0.02	17	0
101	Mining &Mineral Processing	1	0.02	1	0
102	Anesthesiology	1	0.02	38	0
103	Linguistics	1	0.02	0	0
104	Area Studies	1	0.02	0	0
105	Nuclear Science &Technology	1	0.02	12	0
106	Nursing	1	0.02	32	0
107	Obstetrics & Gynecology	1	0.02	7	0
108	Robotics	1	0.02	0	0
109	Transportation	1	0.02	0	0
110	Ethnic Studies	1	0.02	0	0
111	Astronomy & Astrophysics	1	0.02	22	0
C: S_{Title}					
1	Public, Environmental & Occupational Health	845	26.72	15079	17.84
2	Substance Abuse	563	17.81	9904	17.59
3	General & Internal Medicine	451	14.26	4188	9.29
4	Business & Economics	185	5.85	1172	6.34
5	Government & Law	85	2.69	339	3.99
6	History	75	2.37	51	0.68
7	Engineering	67	2.12	147	2.19
8	Health Care Sciences & Services	61	1.93	626	10.26
9	Social Sciences - Other Topics	53	1.68	293	5.53
10	Oncology	51	1.61	286	5.61
11	Respiratory System	44	1.39	73	1.66
12	Psychiatry	41	1.30	713	17.39

13	Science & Technology - Other Topics	33	1.04	156	4.73
14	Computer Science	32	1.01	31	0.97
15	Agriculture	30	0.95	218	7.27
16	Environmental Sciences & Ecology	28	0.89	179	6.39
17	Chemistry	28	0.89	54	0
18	Architecture	25	0.79	1	0
19	Biomedical Social Sciences	21	0.66	480	22.86
20	Toxicology	19	0.60	148	0
21	Sociology	19	0.60	90	0
22	Operations Research & Management Science	19	0.60	65	3.42
23	Arts & Humanities - Other Topics	19	0.60	6	0
24	Area Studies	18	0.57	41	0
25	Pediatrics	16	0.51	227	0
26	Food Science & Technology	16	0.51	3	0
27	Public Administration	16	0.51	32	0
28	Psychology	16	0.51	40	0
29	Anthropology	13	0.41	57	0
30	Literature	12	0.38	2	0
31	Art	12	0.38	1	0
32	Communication	11	0.35	123	0
33	History & Philosophy of Science	11	0.35	22	0
34	Cardiovascular System & Cardiology	10	0.32	53	0
35	Nursing	10	0.32	16	0
36	International Relations	10	0.32	35	0
37	Information Science & Library Science	10	0.32	37	0
38	Pharmacology & Pharmacy	10	0.32	18	0
39	Materials Science	8	0.25	1	0
40	Research & Experimental Medicine	8	0.25	17	2.13
41	Cell Biology	7	0.22	13	0
42	Dance	7	0.22	0	0
43	Mathematics	6	0.19	13	0
44	Dentistry, Oral Surgery & Medicine	6	0.19	12	0
45	Education & Educational Research	6	0.19	32	0
46	Social Issues	6	0.19	168	0
47	Entomology	6	0.19	24	0
48	Theater	6	0.19	4	0
49	Mathematical Methods In Social Sciences	5	0.16	11	2.20
50	Geography	5	0.16	10	0
51	Medical Informatics	5	0.16	66	0
52	Otorhinolaryngology	4	0.13	6	0
53	Telecommunications	4	0.13	5	0
54	Asian Studies	4	0.13	12	0
55	Womens Studies	4	0.13	31	0
56	Water Resources	3	0.09	55	0
57	Energy & Fuels	3	0.09	1	0
58	Infectious Diseases	3	0.09	23	0
59	Plant Sciences	3	0.09	1	0
60	Automation & Control Systems	3	0.09	5	0
61	Meteorology & Atmospheric Sciences	3	0.09	20	0
62	Biotechnology & Applied Microbiology	3	0.09	0	0
63	Urban Studies	3	0.09	5	0
64	Genetics & Heredity	3	0.09	48	0
65	Archaeology	3	0.09	2	0
66	Rehabilitation	3	0.09	2	0
67	Construction & Building Technology	3	0.09	4	0
68	Development Studies	2	0.06	23	0
69	Cultural Studies	2	0.06	3	0
70	Mechanics	2	0.06	0	0
71	Linguistics	2	0.06	2	0
72	Remote Sensing	2	0.06	0	0
73	Music	2	0.06	0	0
74	Medical Ethics	2	0.06	21	0
75	Surgery	2	0.06	7	0
76	Allergy	2	0.06	27	0

77	Dermatology	2	0.06	41	0
78	Geriatrics & Gerontology	2	0.06	24	0
79	Spectroscopy	2	0.06	5	0
80	Nutrition & Dietetics	2	0.06	7	0
81	Legal Medicine	2	0.06	21	10.50
82	Thermodynamics	2	0.06	6	0
83	Polymer Science	1	0.03	35	0
84	Mining & MineralProcessing	1	0.03	1	0
85	Ethnic Studies	1	0.03	3	0
86	Acoustics	1	0.03	0	0
87	Immunology	1	0.03	10	0
88	Sport Sciences	1	0.03	0	0
89	Neurosciences & Neurology	1	0.03	175	0
90	Nuclear Science & Technology	1	0.03	3	0
91	Radiology, Nuclear Medicine &Medical Imaging	1	0.03	3	0
92	Metallurgy & Metallurgical Engineering	1	0.03	0	0
93	Life Sciences & Biomedicine - Other Topics	1	0.03	0	0
94	Microbiology	1	0.03	24	0
95	Film, Radio & Television	1	0.03	0	0
96	Physics	1	0.03	0	0
